# Takeaway Food Consumption, Dietary Inflammatory Index, and Cardiometabolic Risk Factors in US Adults: Findings From NHANES (2009–2018)

**DOI:** 10.1002/fsn3.71316

**Published:** 2025-12-09

**Authors:** Huai Wen, Sijie Li, Marady Hun, Yang Yang, Lei Xiong, Haibo Wen, Lihong Mao, Di Yu, Mingcong Chen, Zisai Wang, Ting C. Zhao, Mingyi Zhao, Qingnan He

**Affiliations:** ^1^ Department of Pediatrics, The Third Xiangya Hospital Central South University Changsha China; ^2^ Pediatrics Department Zhangjiajie People's Hospital Zhangjiajie China; ^3^ Department of Traditional Chinese Geriatric Medicine Xinning Country People's Hospital Shaoyang China; ^4^ Intensive Care Unit Xiangyin People's Hospital Yueyang China; ^5^ College of Computer Science and Electronic Engineering Hunan University Changsha China; ^6^ Cardiovascular and Metabolism Laboratories, Department of Surgery and Plastic Surgery, Rhode Island Hospital Warren Alpert Medical School of Brown University Providence Rhode Island USA

**Keywords:** cardiometabolic risk factors, cardiovascular disease, dietary inflammatory index, mortality, takeaway food consumption

## Abstract

Takeaway food consumption (TFC) has risen and often reflects energy‐dense, nutrient‐poor patterns. The Dietary Inflammatory Index (DII) quantifies diet‐related inflammatory potential. We tested whether higher TFC aligns with higher DII and adverse cardiometabolic risk factors (CRFs) in US adults, and secondarily with all‐cause and heart disease mortality. Data from 8556 NHANES 2009–2018 participants were analyzed. Weighted logistic/linear regression assessed TFC‐DII and TFC‐CRF associations. Weighted Cox models and Kaplan–Meier curves evaluated mortality (via NHANES Linked Mortality File). Exploratory mediation analyses examined indirect effects via DII, cardiometabolic index (CMI), and visceral adiposity index (VAI). Compared with TFC 0–1/week, ≥ 6/week was associated with higher energy‐adjusted DII (*β* = 0.226; 95% CI: 0.061, 0.392), lower HDL (*β* = −1.597; 95% CI: −2.767, −0.426), and higher triglycerides (*β* = 7.980; 95% CI: 1.202, 14.757). Incremental increases in TFC were associated with higher fasting glucose, serum insulin, and insulin resistance. TFC showed no significant mortality associations, whereas higher DII predicted all‐cause mortality (HR = 1.0717; 95% CI: 1.0126, 1.1343). Frequent TFC is linked to a more pro‐inflammatory diet and adverse CRFs. Although TFC alone was not associated with mortality, dietary inflammation predicted increased all‐cause mortality, suggesting a potential long‐term pathway. Reducing high‐frequency TFC and lowering dietary inflammatory potential may improve cardiometabolic health at a population level.

## Graphical abstract

Frequent takeaway food consumption (TFC) is linked to a higher Dietary Inflammatory Index (DII), indicating increased pro‐inflammatory biomarkers. These inflammatory responses are associated with elevated cardiometabolic risk factors (CRFs), including altered blood pressure, lipid profiles, and glucose metabolism. Over time, these adverse changes may contribute to the development of cardiovascular diseases (CVDs).

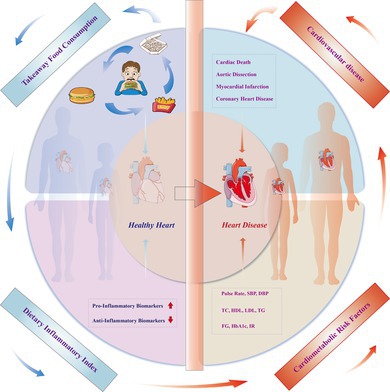

## 1 Introduction

Cardiovascular disease (CVD) imposes a substantial global health and economic burden, with mortality and disability rising over recent decades and projections indicating further growth (Virani et al. 2020; Mortality, G.B.D. and Causes of Death, C 2016). In many settings, CVD‐related deaths exceed those from cancer and chronic lower respiratory diseases (Virani et al. 2020). Our analysis of US trends from 1990 to 2021 (Figure S1), based on Global Burden of Disease (GBD) database, shows a marked rise in CVD deaths and disability‐adjusted life years (DALYs), particularly in the past decade, underscoring the escalating public‐health challenge. Cardiometabolic risk factors (CRFs) are increasingly observed at younger ages, especially in the United States, where their prevalence among youth reflects a complex interplay of genetic, environmental, and lifestyle factors (Arnett et al. 2019; Nichols et al. 2013). Because many CRFs are modifiable, lifestyle approaches, particularly improvement in diet quality, are cost‐effective and scalable strategies for prevention (Leong et al. 2017; Egan et al. 2010).

Global shifts in eating patterns, enabled by internet‐based delivery and economic growth, have contributed to greater consumption of takeaway food (TF) (Nielsen et al. 2002; Smith et al. 2009). Over the past decade, takeaway food consumption (TFC) has become more prevalent among adults—particularly young and middle‐aged—driven by affordability, convenience, pervasive marketing, and time constraints in working households (Smith et al. 2009; Golper et al. 2021; Maimaiti et al. 2018). TF now plays a prominent role in urban, economic, and family life (Maimaiti et al. 2018; Neckerman 2014). Frequent TFC has been associated with CRFs in younger adults, including obesity, hypertension, and dyslipidemia, consistent with energy‐dense, nutrient‐poor dietary patterns low in fruits and vegetables (Smith et al. 2009, 2012). Despite growing public health attention, the multifaceted nature of TFC and its health impacts remain challenging to study (Neckerman 2014). Moreover, evidence linking TFC to diet‐related inflammatory potential quantified by the Dietary Inflammatory Index (DII) in nationally representative United States samples is limited.

Chronic low‐grade inflammation and immune activation are key contributors to CVD development, linking CVD risk to nutrient intake and inflammatory responses (Hansson 2005; Mazidi et al. 2018). Diet is a major environmental determinant of systemic inflammation, and poor dietary patterns drive elevations in inflammatory biomarkers (Hariharan et al. 2022). For example, Western dietary patterns high in fat are associated with increased inflammatory biomarkers (Khayyatzadeh et al. 2018), whereas Mediterranean‐style patterns, rich in vegetables and fish, reduce inflammation and lower cardiometabolic risks (Ahluwalia et al. 2013). Recognizing that pro‐inflammatory diets increase the body's inflammatory burden (Johansson‐Persson et al. 2014), Shivappa and colleagues developed the DII to quantify a diet's inflammatory potential (Shivappa et al. 2014). Higher DII scores, reflecting more inflammatory diets, have been associated with increased CVD incidence and mortality (Ruiz‐Canela et al. 2016). Mechanistically, diet‐induced inflammation promotes endothelial dysfunction, plaque vulnerability, and thrombosis, providing a biologic link to both cardiovascular and cerebrovascular events (Pacinella et al. 2022; Li et al. 2020; Libby 2021). TFC, as part of unhealthy dietary patterns, tends to elevate systemic inflammation, and frequent consumption has been associated with adverse outcomes, such as CRFs (Saraf et al. 2022). Diet is a modifiable factor that plays a central role in CVD progression (Saraf et al. 2022; Collaborators 2019).

Young and middle adulthood are critical stages for adopting and sustaining healthy behaviors that prevent CRFs (Gordon‐Larsen et al. 2010). Achieving and maintaining ideal cardiovascular health across adulthood—including midlife and older age—is essential to lowering lifetime cardiovascular risk (Shay et al. 2013). Accordingly, prevention strategies increasingly target the full adult life course, aligned with the American Heart Association's (AHA) Strategic Impact Goals that emphasize primordial prevention to avert the onset of CVD risk factors (Lloyd‐Jones et al. 2010). Despite growing awareness of unhealthy dietary patterns, diet‐related inflammation—and its contribution to CVD—remains insufficiently addressed, in part because research on dietary inflammation and CRFs has disproportionately focused on older adults (Saraf et al. 2022; Odegaard et al. 2012). Meanwhile, TFC is rising among adults, especially younger and middle‐aged groups, underscoring the need for population‐level approaches that improve diet within real‐world food environments (Pereira et al. 2005).

Descriptive analyses of US CVD deaths and DALYs (1990–2021) suggest an inflection around 2010, coinciding with global growth in app‐based food delivery; this temporal overlap does not imply causation (Li et al. 2022). Against this background, we treat TFC as a modifiable, policy‐relevant dietary behavior. Using NHANES 2009–2018, we aimed to (i) quantify the association between TFC frequency and energy‐adjusted DII; (ii) evaluate associations of TFC with key CRFs; and (iii) test whether DII—secondarily, the cardiac metabolic index (CMI) and the visceral adiposity index (VAI)—mediates TFC–CRF relationships in a nationally representative adult sample. Secondly, using the linked mortality files from NHANES, we analyzed the associations between ultra‐processed food consumption, the dietary inflammatory index, and all‐cause and heart disease mortality among 8556 participants through a survey‐weighted Cox model. We hypothesized that higher TFC would align with higher DII and adverse CRFs, that DII (and secondarily CMI/VAI) would partially mediate these associations, and that DII—given its proximity to inflammatory pathways—would show positive associations with mortality, whereas TFC–mortality associations might be attenuated by exposure measurement error and cause‐of‐death categorization in public‐use files.

## 2 Methods

### 2.1 Measurements and Sample

This study used 10 years of cross‐sectional NHANES data from 2009 to 2018, which collects biennial health and nutrition data from a nationally representative sample of the US population. NHANES employs a multistage probability sampling design, selecting participants based on geographic and minority strata. Participants were interviewed at home and invited to a mobile examination center for assessments, dietary recall, and blood sample collection (Centers for Disease Control and Prevention 2022). All data were collected following NHANES protocols. Detailed questionnaires and protocols are available elsewhere.

**FIGURE 1:**
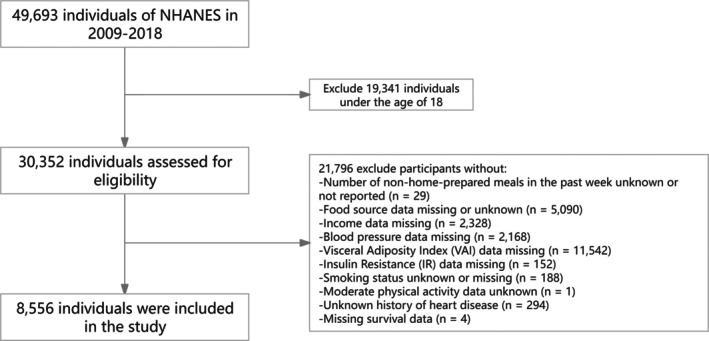
Study flow chart.

The NHANES 2009–2018 dataset included 49,693 participants. In this analysis, 8556 people were included in the study. First, 19,341 participants under the age of 18 were excluded. Subsequently, an additional 21,796 participants were excluded due to missing data on key variables, including food sources (*n* = 5090), the Visceral Adiposity Index (VAI) (*n* = 11,542), income (*n* = 2328), blood pressure (*n* = 2168), smoking status (*n* = 188), history of heart disease (*n* = 294), the number of non‐home‐prepared meals (*n* = 29), Insulin Resistance (IR) (*n* = 152), survival data (*n* = 4), and moderate physical activity (*n* = 1) (The detailed screening process is shown in Figure 1). Only participants with complete data on all 11 CRFs, TFC frequency, and DII scores were included in the analysis. The study was approved by the CDC and NCHS Ethical Review Board, and all participants provided written informed consent (https://www.cdc.gov/nchs/nhanes/irba98.htm). For minors, consent was obtained from parents or guardians, while adults provided their own consent.

### 2.2 Sociodemographic Characteristics

Sociodemographic data, including age, gender, race/ethnicity, education level, marital status, employment status, and household income, were gathered during home interviews. Participants were classified by race/ethnicity as White, Black, and Other races. Education was grouped into three levels: less than high school, high school graduate or equivalent, and college or higher.

We identified a priori confounders based on established associations with both exposure and outcome variables (Virani et al. 2020; Arnett et al. 2019). These included BMI categories (underweight: < 18.5 kg/m^2^, normal weight: 18.5–24.99 kg/m^2^, overweight: 25–29.99 kg/m^2^, and obesity: ≥ 30 kg/m^2^) (World Health Organization 2000), waist circumference (Saydah et al. 2013), and smoking status (never: fewer than 100 lifetime cigarettes; smoker: at least 100 lifetime cigarettes) (Strozyk et al. 2018). We defined the daily above‐medium physical activity level as ideal (meeting or exceeding recommendations); otherwise, it was classified as non‐ideal (below recommendations) (Ryu et al. 2020).

### 2.3 Definition of TFC Frequency

TF typically refers to items that can be quickly purchased from physical locations or online platforms, such as fried chicken, burgers, fries, pizza, and other fast food options. In this study, TFC was defined as food obtained from fast food or pizza places, meals on wheels, vending machines, coffee pots or snack trays, mail orders, street vendors, or vending trucks, as recorded through 24‐h dietary recall data. TFC was measured with two questions: “During the past 7 days, how many meals did you get that were prepared away from home in places such as restaurants, fast food places, food stands, grocery stores, or from vending machines?” and “Where did you get this/most of the ingredients for this (including fast food/pizza place, meals on wheels, vending machine, common coffee pot or snack tray, mail order purchase, or street vendor, vending truck)?” Based on the distribution of responses, TFC frequency was categorized into four groups: Never (0–1 times/week), Low (2–3 times/week), Moderate (4–5 times/week), and High (≥ 6 times/week).

### 2.4 Dietary Inflammatory Index (DII)

The DII, developed by Shivappa, is a tool designed to assess the inflammatory potential of an individual's diet, based on a thorough review of peer‐reviewed literature (Shivappa et al. 2014). A total of 1943 research articles, published between 1950 and 2010, were analyzed, linking 45 dietary components, including nutrients, foods, and flavonoids, to inflammatory markers. Each component was assigned a score based on its impact on four pro‐inflammatory biomarkers (IL‐1β, IL‐6, TNF‐α, and C‐reactive protein [CRP]) and two anti‐inflammatory biomarkers (IL‐4 and IL‐10). A score of +1 indicated an increase, −1 indicated a decrease, and 0 indicated no effect. Dietary intake data were standardized against global daily averages, generating Z‐scores and centered percentiles. The global reference data were derived from 11 regions around the world (Shivappa et al. 2014). A detailed breakdown of DII for the NHANES 2009–2018 study is provided in Table S1.

Dietary data for NHANES 2009–2018 participants were collected through 24‐h dietary recall interviews. Of the 45 DII food parameters, 26 were available in NHANES, including energy, proteins, Carbohydrate, Dietary fiber, total fat, Total saturated fatty acids, total monounsaturated fatty acids (MUFAs), total polyunsaturated fats (PUFAs), Cholesterol, vitamins (A, B1, B2, B6, B12, C, D, E), Beta‐carotene, Niacin, Folic acid, iron, magnesium, zinc, selenium, alcohol, and caffeine. Each parameter's centered percentile was multiplied by its respective inflammatory effect score, and the resulting values were summed to calculate an overall DII score for each participant. Higher (more positive) scores indicated a more pro‐inflammatory diet, while lower scores reflected a more anti‐inflammatory diet (Shivappa et al. 2014). To account for total energy intake, DII was adjusted per 1000 cal consumed, using a global calorie‐adjusted reference database. Even if fewer than 30 parameters are available, DII scores can still be calculated (Li et al. 2020).

### 2.5 Cardiometabolic Risk Factors (CRFs) Components

CRFs were assessed during the 2009–2018 NHANES interviews and examinations. These factors included pulse rate (beats per minute), systolic blood pressure (SBP), diastolic blood pressure (DBP), total cholesterol (TC) (mg/dL), high‐density lipoprotein (HDL) (mg/dL), low‐density lipoprotein (LDL) (mg/dL), triglycerides (TG) (mg/dL), fasting glucose (FG) (mmol/L), glycated hemoglobin (HbA1c) (%), serum insulin (μU/mL), insulin resistance (IR), cardiac metabolic index (CMI) and visceral adiposity index (VAI). Blood pressure measurements were taken at the mobile examination center (MEC) by certified BP examiners trained through Shared Care Research and Education Consulting. After participants rested for 5 min, three consecutive blood pressure readings were recorded, with a fourth reading if needed. Insulin resistance (IR) was calculated using the HOMA‐IR formula: fasting insulin (μU/mL) × fasting glucose (mmol/L)/22.5. The cardiac metabolic index (CMI) calculation formula is based on the LAP formula modification: triglycerides (mmol/L)/high‐density lipoprotein cholesterol (mmol/L) × waist height ratio. Formula for calculating visceral fat index (VAI): Male: VAI = waist circumference (cm)/(39.68 + 1.88 × body mass index [kg/m^2^]) × triglycerides (mmol/L)/1.03 × 1.31/high‐density lipoprotein cholesterol (mmol/L); female: VAI = waist circumference (cm)/(36.58 + 1.89 × body mass index [kg/m^2^]) × triglycerides (mmol/L)/0.81 × 1.52/high‐density lipoprotein cholesterol (mmol/L). Blood samples were processed, stored, and sent to the University of Minnesota for analysis. Detailed procedures for the collection, processing, and measurement of TC, HDL, LDL, TG, FG, HbA1c, and serum insulin are available in the NHANES Laboratory Procedures Manual (LPM) (https://wwwn.cdc.gov/nchs/nhanes/search/datapage.aspx?Component=Laboratory).

### 2.6 All‐Cause and Heart Disease Mortality

We used the ID matching method to link the participants in the NHANES in the United States from 2009 to 2018 with the Linked Mortality File (LMF) publicly available from the National Center for Health Statistics in 2019 to determine mortality. This file is linked to the National Death Index as of December 31, 2018. All‐cause mortality was defined as death from any cause before the end of follow‐up (LMF variable MORTSTAT = 1). Heart disease mortality was defined according to the underlying cause of death recoded from the variable UCOD_LEADING in the LMF (UCOD_113 codes 54–68); since the LMF only provides grouped causes of death, we reported “heart disease” rather than the broader category of cardiovascular diseases. The variable for follow‐up time is PERMTH_EXM, calculated in person‐months, starting from the date of the Mobile Examination Center (MEC) examination and ending on the date of death or December 31, 2018 (whichever came first), and was calculated and converted to person‐years (PERMTH_EXM/12). We restricted the survival analysis to adults aged 18–80 years with valid mortality data linkage and excluded participants with missing LMF variables. All survival models incorporated the NHANES complex survey design (weights, stratification, and primary sampling units).

### 2.7 Statistical Analysis

All analyses were performed using R software (version 4.3.3). Because NHANES employs a complex, multistage probability sampling design with oversampling of specific subgroups, survey weights were applied to obtain nationally representative estimates. Continuous variables were summarized as mean ± SD or median (IQR), and categorical variables as weighted percentages. Group comparisons used the *t*‐test or Mann–Whitney test for continuous variables and the *χ* ^2^ test for categorical variables. Associations between TFC frequency, DII, and CRFs were evaluated using survey‐weighted logistic and linear regression models, adjusted for potential confounders and stratified by sex and age. For mortality analyses, person‐time was calculated from the NHANES examination date to the date of death or censoring (December 31, 2018). Mortality rates were expressed as events per 1000 person‐years. Survey‐weighted Cox proportional hazards models estimated hazard ratios (HRs) and 95% confidence intervals (CIs), and the proportional hazards assumption was verified using Schoenfeld residuals. Kaplan–Meier curves were plotted to visualize survival probabilities across TFC categories. Subgroup and sensitivity analyses examined robustness across strata of sex, age, BMI, smoking status, and physical activity. To explore underlying mechanisms, we applied a structural equation modeling (SEM) framework to quantify the total, direct, and indirect effects of TFC on mortality through DII, CMI, and VAI, with bootstrap‐derived 95% CIs.

## 3 Results

### 3.1 Description of Baseline Characteristics

Table 1 summarizes the weighted demographic data and characteristics of study participants across different DII groups. The analysis included 8556 individuals aged 18–80 years, with a gender distribution of 49.59% males and 50.41% females. Younger participants were found to have a higher frequency of TFC (*p* < 0.001).

**TABLE 1:** Baseline characteristics of the study population grouped by DII.

Characteristics	Overall	TFC frequency				*p*
		Q1 (< 2)	Q2 (2–4)	Q3 (4–6)	Q4 (≧ 6)	
*N* (%)	8556 (100%)	3218 (37.61%)	2453 (28.67%)	1324 (15.47%)	1561 (18.24%)	
Age (years)	47.54 (46.90, 48.17)	51.73 (50.90, 52.56)	47.78 (46.75, 48.80)	45.38 (44.15, 46.61)	42.99 (41.87, 44.12)	< 0.0001
Sex (%)						
Male	49.59 (48.18, 51.00)	43.02 (40.29, 45.80)	46.00 (43.59, 48.43)	53.09 (49.48, 56.67)	60.88 (57.56, 64.09)	< 0.0001
Female	50.41 (49.00, 51.82)	56.98 (54.20, 59.71)	54.00 (51.57, 56.41)	46.91 (43.33, 50.52)	39.12 (35.91, 42.44)	
Race (%)						
White	68.52 (65.43, 71.45)	63.95 (60.09, 67.63)	70.42 (66.53, 74.03)	71.30 (67.43, 74.87)	70.23 (66.33, 73.85)	< 0.0001
Black	9.78 (8.39, 11.37)	10.08 (8.46, 11.98)	9.15 (7.45, 11.19)	10.25 (8.49, 12.32)	9.79 (7.95, 12.00)	
Other	21.71 (19.34, 24.28)	25.97 (22.80, 29.40)	20.43 (17.51, 23.69)	18.45 (15.76, 21.49)	19.99 (17.16, 23.15)	
Education attainment (%)						
Less than high school	14.64 (13.28, 16.11)	21.98 (19.66, 24.50)	12.93 (11.22, 14.87)	11.53 (9.76, 13.58)	8.99 (7.24, 11.13)	< 0.0001
High school	22.35 (20.78, 23.99)	24.21 (21.87, 26.72)	22.19 (19.80, 24.79)	20.96 (18.06, 24.20)	21.02 (18.10, 24.27)	
Higher than high school	63.02 (60.61, 65.36)	53.81 (50.43, 57.15)	64.87 (61.62, 68.00)	67.50 (63.81, 70.99)	69.99 (66.37, 73.37)	
Poverty income ratio (%)	2.97 (2.88, 3.06)	2.54 (2.42, 2.66)	3.07 (2.96, 3.19)	3.15 (3.02, 3.29)	3.31 (3.18, 3.43)	< 0.0001
Height (cm)	169.05 (168.74, 169.36)	167.00 (166.51, 167.48)	168.64 (168.16, 169.11)	170.40 (169.71, 171.10)	171.41 (170.66, 172.15)	< 0.0001
Weight (Kg)	83.40 (82.70, 84.10)	80.68 (79.39, 81.96)	83.10 (82.04, 84.16)	85.97 (84.54, 87.39)	85.60 (84.24, 86.96)	< 0.0001
Waist circumference (cm)	99.83 (99.23, 100.43)	99.56 (98.64, 100.49)	99.67 (98.81, 100.52)	100.63 (99.48, 101.79)	99.78 (98.69, 100.87)	0.4027
BMI (kg/m^2^)	29.11 (28.86, 29.35)	28.85 (28.45, 29.25)	29.16 (28.78, 29.54)	29.16 (28.78, 29.54)	29.04 (28.65, 29.42)	0.1522
BMI category (%)						
Underweight	1.49 (1.19, 1.87)	1.69 (1.14, 2.50)	0.96 (0.61, 1.49)	1.69 (0.93, 3.05)	1.77 (1.15, 2.72)	0.3044
Healthy weight	27.64 (26.07, 29.27)	29.25 (26.85, 31.77)	28.54 (25.92, 31.31)	25.17 (21.19, 29.62)	26.16 (23.00, 29.57)	
Overweight	33.12 (31.85, 34.41)	33.70 (31.63, 35.84)	32.15 (29.38, 35.05)	33.90 (30.23, 37.78)	32.94 (29.85, 36.18)	
Obese	37.75 (36.21, 39.32)	35.36 (32.65, 38.16)	38.36 (35.44, 41.37)	39.24 (35.90, 42.68)	39.14 (36.18, 42.17)	
Pulse (times)	71.43 (71.01, 71.84)	71.77 (71.05, 72.50)	71.31 (70.76, 71.85)	71.35 (70.58, 72.12)	71.15 (70.32, 71.98)	0.6119
SBP (mmHg)	120.98 (120.42, 121.54)	122.64 (121.72, 123.57)	121.08 (120.22, 121.94)	120.29 (119.31, 121.26)	119.06 (118.24, 119.88)	0.0017
DBP (mmHg)	69.67 (69.13, 70.21)	68.97 (68.23, 69.70)	69.44 (68.71, 70.17)	70.43 (69.61, 71.24)	70.35 (69.55, 71.15)	< 0.0001
HDL (mg/dL)	54.30 (53.74, 54.86)	55.77 (54.86, 56.69)	54.32 (53.28, 55.35)	53.92 (52.70, 55.14)	52.50 (51.52, 53.48)	0.0003
LDL (mg/dL)	113.47 (112.32, 114.63)	114.89 (113.11, 116.67)	113.15 (111.52, 114.78)	112.71 (110.80, 114.62)	112.50 (110.02, 114.98)	0.1484
TC (mg/dL)	191.52 (190.02, 193.01)	193.84 (191.63, 196.06)	191.67 (189.52, 193.81)	190.45 (187.98, 192.91)	188.87 (185.81, 191.93)	0.0171
TG (mg/dL)	120.69 (117.43, 123.95)	117.11 (113.09, 121.12)	124.12 (118.58, 129.66)	121.66 (115.23, 128.10)	120.40 (113.99, 126.81)	0.1994
FG (mg/dL)	5.92 (5.86, 5.97)	5.99 (5.91, 6.08)	5.89 (5.79, 5.98)	5.82 (5.74, 5.90)	5.93 (5.83, 6.03)	0.0322
HbAlc (%)	5.64 (5.61, 5.67)	5.72 (5.68, 5.77)	5.64 (5.59, 5.68)	5.57 (5.51, 5.62)	5.58 (5.54, 5.63)	< 0.0001
Serum insulin (uU/mL)	13.08 (12.63, 13.53)	12.78 (12.15, 13.42)	12.49 (11.92, 13.07)	14.02 (12.98, 15.06)	13.54 (12.59, 14.50)	0.0276
Insulin resistance	3.73 (3.56, 3.89)	3.78 (3.51, 4.06)	3.49 (3.28, 3.70)	3.91 (3.56, 4.27)	3.82 (3.47, 4.16)	0.0409
CMI	1.66 (1.58, 1.73)	1.57 (1.49, 1.66)	1.71 (1.59, 1.83)	1.69 (1.54, 1.84)	1.68 (1.54, 1.82)	0.2036
VAI	4.43 (4.26, 4.61)	4.32 (4.12, 4.52)	4.63 (4.33, 4.93)	4.43 (4.06, 4.79)	4.34 (4.01, 4.67)	0.3525
Smoking status						
Yes	44.93 (43.03, 46.84)	48.97 (46.30, 51.66)	43.34 (40.28, 46.46)	46.04 (42.28, 49.84)	40.38 (36.54, 44.34)	0.0007
No	55.07 (53.16, 56.97)	51.03 (48.34, 53.70)	56.66 (53.54, 59.72)	53.96 (50.16, 57.72)	59.62 (55.66, 63.46)	
Vigorous work activity						
Yes	42.78 (40.95, 44.64)	37.82 (35.15, 40.58)	45.36 (42.74, 48.01)	47.12 (43.38, 50.90)	42.89 (39.54, 46.30)	< 0.0001
No	57.22 (55.36, 59.05)	62.18 (59.42, 64.85)	54.64 (51.99, 57.26)	52.88 (49.10, 56.62)	57.11 (53.70, 60.46)	
DII	1.01 (0.93, 1.09)	1.05 (0.93, 1.16)	1.02 (0.90, 1.14)	0.95 (0.80, 1.10)	0.98 (0.84, 1.11)	0.6019

*Note:* 95% confidence interval for continuous variables: *p* value was by survey‐weighted linear regression. 95% confidence intervals for the categorical variables: *p* value was calculated by weighted chi‐squared test.

Abbreviations: BMI, body mass index; CMI, cardiac metabolic index; DBP, diastolic blood pressure; DII, dietary inflammatory index; FG, fasting glucose; HbAlc, glycated hemoglobin; HDL, high‐density lipoprotein; IR, Insulin resistance; LDL, low‐density lipoprotein; SBP, systolic blood pressure; TC, total cholesterol; TG, triglycerides; VAI, visceral fat index.

Significant gender differences in DII scores were found (*p* < 0.001), with more males in the highest TFC frequency category (Q4 ≥ 6) at 60.88%, compared to 39.12% of females. Within TFC subgroups, there were notable differences in race, education, income, height, weight, blood pressure, cholesterol, glucose, insulin, smoking, and physical activity (*p* < 0.05) (Table 1). Participants with higher TFC frequencies generally had lower HDL, total cholesterol, fasting glucose, and smoking rates, but higher income, weight, height, insulin, and insulin resistance. Interestingly, lower TFC groups (Q1 < 2; Q2: 2–4) had higher DII values (1.05 and 1.02), while higher TFC groups (Q3: 4–6; Q4 ≥ 6) had lower DII values (0.95 and 0.98) (Table 1).

Table S1 summarizes the weighted statistical data and dietary characteristics of participants by TFC frequency. Most dietary factors increased with higher TFC frequency, with significant baseline differences for most inflammatory factors, except for fiber, vitamin A, beta‐carotene, vitamin C, and caffeine (*p* < 0.05). Notably, saturated and polyunsaturated fatty acids were positively associated with higher TFC frequency (*p* < 0.0001), while DII scores for vitamin B6, E, magnesium, zinc, selenium, and alcohol decreased with increasing TFC frequency.

### 3.2 Associations Between TFC Frequency, DII With CRFs

**FIGURE 2:**
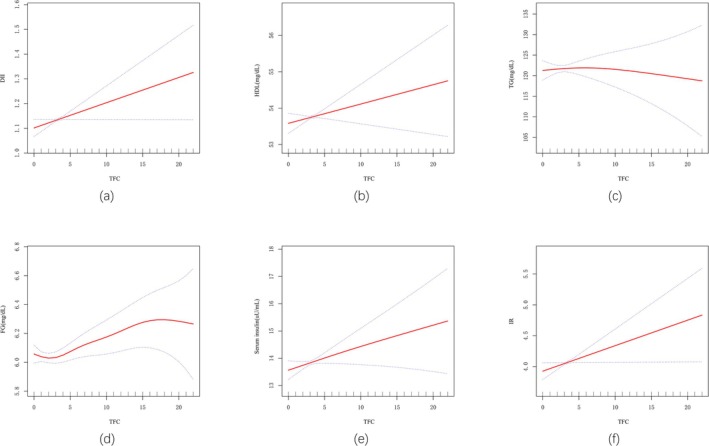
The associations between TFC and key health indicators: (a) TFC and DII; (b) TFC and HDL; (c) TFC and TG; (d) TFC and FG; (e) TFC and serum insulin; (f) TFC and IR. The solid red line represents the smoothed curve fit between variables, while the blue bands indicate the 95% confidence interval. All models are adjusted for age, sex, race, educational attainment, poverty‐income ratio, weight, height, smoking status, and physical activity. DII, Dietary Inflammatory Index; FG, fasting glucose; HDL, high‐density lipoprotein; IR, insulin resistance; TFC, takeaway food consumption; TG, triglycerides.

As shown in Table 2, weighted TFC was significantly associated with DII in the generalized linear regression model (*p* = 0.0117). In Model 3, stratified by TFC frequency, DII was significantly higher in Q4 compared to Q1 (*β* = 0.226; 95% CI: 0.061, 0.392; *p* = 0.0095), with a significant upward trend observed (*p* = 0.0142). Tables S3 and S4, and Figure 2 indicate no significant association between TFC and HDL or TG when treated as a continuous variable. However, stratified analysis revealed a significant decrease in HDL (Q4 group, Model 3: *β* = −1.597; 95% CI: −2.767, −0.426; *p* = 0.0095) (Table S2) and a significant increase in TG (Q4 group, Model 3: *β* = 7.980; 95% CI: 1.202, 14.757; *p* = 0.0243) (Table S3).

**TABLE 2:** Association of TFC and DII in 8556 individuals aged 18–80 years: NHANES 2009–2018.

	Model 1		Model 2		Model 3	
	*β* (95% CI)	*p*	*β* (95% CI)	*p*	*β* (95% CI)	*p*
TFC	−0.004 (−0.018, 0.011)	0.6156	0.013 (−0.002, 0.027)	0.0878	0.019 (0.005, 0.034)	0.0117
Q1 (0–1)	0		0		0	
Q2 (2–3)	−0.027 (−0.173, 0.120)	0.7238	0.060 (−0.086, 0.205)	0.4235	0.115 (−0.029, 0.259)	0.1221
Q3 (4–5)	−0.102 (−0.267, 0.064)	0.2327	0.039 (−0.129, 0.207)	0.6530	0.101 (−0.066, 0.268)	0.2388
Q4 (≧ 6)	−0.070 (−0.238, 0.098)	0.4165	0.144 (−0.019, 0.308)	0.0882	0.226 (0.061, 0.392)	0.0095
*p* for trend	0.2826		0.1170		0.0142	

*Note:* Model 1: None; Model 2: model adjust for: adjust for Age, Sex, Race, Education attainment; Model 3: model adjust for: adjust for Age, Sex, Race, Education attainment, Poverty income ratio, Weight, Height, Smoking status, Physical activity.

Abbreviations: DII, dietary inflammatory index; TFC, takeout food consumption.

In all models, TFC was significantly associated with FG (Table S4, Figure 2). In the unadjusted Model 1, no correlation was observed between TFC and FG (*β* = −0.006; 95% CI: −0.017, 0.005; *p* = 0.3120). However, after adjusting for age, gender, race, and education, a significant positive association emerged (Model 2: *β* = 0.012; 95% CI: 0.001, 0.023; *p* = 0.0312), which persisted after full adjustment for all covariates (Model 3: *β* = 0.012; 95% CI: 0.002, 0.023; *p* = 0.0263), confirming an upward trend in FG with increasing TFC frequency.

Table S5 and Figure 2 highlight the significant association between TFC frequency and serum insulin. In Models 2 and 3, TFC consistently showed a positive correlation with serum insulin, even after adjusting for age, gender, race, education, income, and physical activity (Model 3: *β* = 0.093; 95% CI: 0.013, 0.173; *p* = 0.0263). The trend test further supported this increase (*p* = 0.0173), with serum insulin levels in higher TFC groups (Q3 and Q4) significantly elevated compared to Q1, particularly in Q4 (*β* = 1.010; 95% CI: −0.002, 2.023; *p* = 0.0548). For IR, no significant association was observed in Models 1 and 2. However, a positive correlation was identified in Model 3 (*β* = 0.034; 95% CI: 0.001, 0.067; *p* = 0.0489) (Table S6, Figure 2). In addition, we examined the associations between TFC and Pulse, SBP, DBP, TC, LDL, and HbA1c; however, none of these results reached statistical significance (Table S8).

### 3.3 Subgroup Analysis

Subgroup analyses stratified by age and gender, as depicted in Figures S2 and S3, show no significant associations between TFC and FG, HDL, TG, serum insulin, or IR within individual age groups, and interaction effects across age stratifications were not statistically significant. However, a significant positive association was observed between TFC and DII, particularly in the 60–80 age group (*β* = 0.046; 95% CI: 0.009, 0.082; *p* = 0.0173) (Figure S2).

In gender‐stratified analyses, females exhibit a more pronounced increase in FG levels with higher TFC compared to males (*β* = 0.011; 95% CI: 0.003, 0.019; *p* = 0.0086) (Figure S3). Additionally, serum insulin levels show a significant positive association with TFC in females (*β* = 0.167; 95% CI: 0.047, 0.287; *p* = 0.0082), an effect not observed in males. Similarly, IR levels significantly increase with rising TFC in females (*β* = 0.066; 95% CI: 0.015, 0.117; *p* = 0.0144), indicating a stronger metabolic response to TFC in this subgroup. Notably, TFC is also significantly associated with DII in gender stratification, particularly among males (*β* = 0.019; 95% CI: 0.001, 0.038; *p* = 0.0428) (Figure S6).

To further explore the relationship between DII and CRFs, generalized additive models combined with smooth curve fitting techniques were employed. The results are presented in Figures S4 and S5, illustrating age‐ and sex‐stratified associations with key CRFs.

### 3.4 Survival Analysis

During the follow‐up period of 51,937.50 person‐years, a total of 554 all‐cause deaths were recorded, including 128 deaths attributed to CVD (Table S7). To assess the independent impact of TFC on mortality rates, three models were developed, with hazard ratios (HR) and 95% confidence intervals (CI) detailed in Table S7. In the fully adjusted model (Model 3), although the associations did not reach statistical significance (*p* = 0.8207 and *p* = 0.5179, respectively), a 1‐unit increase in TFC was linked to a 0.5% rise in all‐cause mortality risk and a 2.6% rise in CVD mortality risk.

TFC was categorized into quartiles to analyze its impact on mortality. Participants in the Q3 and Q4 groups showed higher HRs for cardiovascular mortality compared to those in the Q1 group, with HRs of 1.2443 (95% CI: 0.5975, 2.5913) and 1.1022 (95% CI: 0.5189, 2.3414), respectively (Table S7). However, these results were not statistically significant. Kaplan–Meier survival curves (Figure S6) show that the all‐cause mortality and cardiovascular mortality in the group with the highest TFC frequency are higher than those in the remaining TFC frequency groups.

Subgroup analyses (Figures S7 and S8) further examined the relationships between TFC and both all‐cause and heart disease mortality across different categories of gender, race, educational attainment, poverty income ratio, and smoking status. No statistically significant associations were identified in any subgroup. However, slight variations in HRs were observed across subgroups, with higher TFC frequencies (Q3 and Q4) generally showing increased HRs for mortality.

**FIGURE 3:**
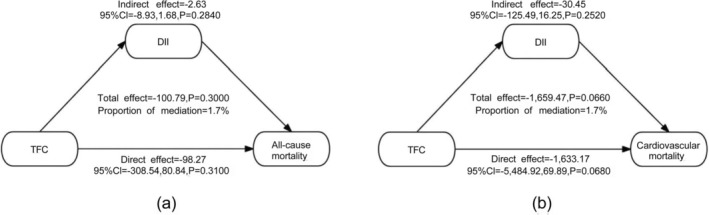
Analysis of the mediating role of DII in the relationship between CMI and all‐cause mortality (a), Analysis of the mediating role of DII in the relationship between CMI and cardiovascular mortality (b). CMI, cardiac metabolic index; DII, Dietary Inflammatory Index.

Figure S9 illustrates the relationships between DII, CMI, VAI, and mortality outcomes based on Cox regression analysis. In the all‐cause mortality group, after adjusting for all potential confounders, DII was significantly associated with an increased risk of mortality (HR = 1.0717, 95% CI: 1.0126, 1.1343; *p* = 0.0168). In the heart disease mortality group, although the association was not statistically significant, DII showed a positive trend with mortality risk (HR = 1.1213, 95% CI: 0.9903, 1.2697; *p* = 0.0708). Figure 3 reveals that DII mediated 1.7% of the association between TFC and all‐cause mortality and 1.7% of the association between TFC and heart disease mortality, with none of the mediation effects reaching statistical significance.

## 4 Discussion

This nationally representative analysis of 8556 US adults shows three main findings. First, higher TFC aligned with higher energy‐adjusted DII. Second, higher TFC is related to an unfavorable cardiometabolic profile—lower HDL and higher triglycerides, fasting glucose, serum insulin, and IR. Third, in the survival study, we found that there was a decreasing trend in survival rate when the TFC level was relatively high; however, survey‐weighted Cox models showed no statistically significant associations with all‐cause or heart disease mortality. In contrast, higher DII predicted all‐cause mortality and showed a positive trend for heart disease mortality, while DII mediated only a small, non‐significant fraction (≈1.7%) of the TFC–mortality association. These results support early, population‐level strategies to curb high‐frequency TFC and reduce dietary inflammatory potential in order to improve cardiometabolic health.

Most individuals enter adulthood with ideal cardiovascular health (Roger et al. 2012), but early CRFs increase late‐life CVD risk (Laitinen et al. 2012; Juonala et al. 2011). Although clinical CVD is more common in older adults, lipid disorders, diabetes, and obesity are rising among younger populations (Third Report of the National Cholesterol Education Program (NCEP) Expert Panel on Detection, Evaluation, and Treatment of High Blood Cholesterol in Adults (Adult Treatment Panel III) Final Report 2002; Steinberger et al. 2009). The persistence of these CRFs underscores the need for early prevention. However, research has largely focused on older adults, with limited attention to younger groups (Franks et al. 2010; He et al. 2009). Our descriptive analysis of US CVD deaths and DALYs (1990–2021) suggests an inflection around 2010 (Figure S1), coinciding with the rise in TF delivery; this temporal overlap does not imply causation (Maimaiti et al. 2018; Li et al. 2022). Given that CRFs often originate in adolescence or early adulthood (Chen et al. 2007) and that healthy lifestyles are central to primordial prevention (Leong et al. 2017; Egan et al. 2010), we hypothesized that higher TFC would be associated with higher DII and a less favorable CRF profile—particularly in younger adults. Our findings support this hypothesis and highlight the importance of early dietary interventions to reduce long‐term CVD risk.

The observed association between higher TFC and higher DII suggests that frequent intake of energy‐dense, fat‐, sugar‐, and sodium‐rich takeaway foods–often low in fruits, vegetables, fiber, and key micronutrients—is associated with a greater systemic inflammation load (Pereira et al. 2005). Diet‐related inflammation is a key driver of vascular disease: pro‐inflammatory patterns correlate with CRP, IL‐6, and TNF‐α and track with atherosclerotic progression (Aroke et al. 2020). A meta‐analysis indicates that each 1‐point increase in DII corresponds to about an 8% higher risk of CVD and mortality (Shivappa et al. 2018). Mechanistically, chronic low‐grade inflammation can impair endothelial function, increase plaque vulnerability, and promote thrombosis, providing a biologic link between pro‐inflammatory dietary patterns and both cardiovascular and cerebrovascular events (Pacinella et al. 2022; Li et al. 2020; Libby 2021). In line with this evidence (Smith et al. 2012; Saraf et al. 2022; Odegaard et al. 2012), our findings that higher TFC align with higher DII and an adverse CRF profile support dietary strategies that lower inflammatory potential to reduce long‐term cardiovascular risk.

While systemic inflammation is a recognized contributor, the specific pathways linking TFC to preclinical CVD are not fully delineated. Beyond inflammation (Hansson 2005; Li et al. 2020), plausible mechanisms include the gut‐heart axis (Bianchi et al. 2022) and oxidative stress (Devaraj et al. 2008). Microbial metabolites and inflammatory mediators can enter the circulation and modulate vascular and myocardial function, supporting bidirectional gut‐heart communication (Kamo et al. 2017). We therefore hypothesize that pro‐inflammatory TFC patterns may worsen CRFs—particularly in younger adults—by amplifying gut‐derived immune signaling and oxidative stress. These mechanistic considerations, while outside the scope of causal inference in the present study, reinforce the rationale for early dietary improvements to mitigate long‐term cardiometabolic risk.

Higher TFC was associated with an adverse cardiometabolic profile—higher triglycerides, fasting glucose, serum insulin, and IR, alongside lower HDL—consistent with an atherogenic lipid milieu (Mazidi et al. 2018; Saraf et al. 2022). Kaplan–Meier curves showed directionally lower survival at higher TFC levels; however, in survey‐weighted Cox models, the associations with all‐cause and heart disease mortality were not statistically significant. Mediation analyses indicated that DII accounted for approximately 1.7% of the TFC—mortality association, and this indirect effect was not significant. In sex‐stratified analyses, associations with fasting glucose, serum insulin, and IR appeared stronger in females, whereas DII associations were more evident in males; these subgroup patterns are interpreted cautiously and only when interaction tests are significant. Overall, while the estimated clinical impact is modest, the evidence implicates high‐frequency TFC and diet‐related inflammation in adverse cardiometabolic profiles, with particular relevance for younger adults.

These findings support reducing unhealthy TFC, particularly among younger adults (Neckerman 2014; Saraf et al. 2022). Given modern time constraints and limited cooking skills that increase reliance on ready‐to‐eat meals, interventions should pair education with environmental changes that make healthier options easy to choose (Odegaard et al. 2012; Powell and Nguyen 2013). Public‐health efforts ought to raise awareness of the cardiometabolic risks of high‐frequency TFC and promote anti‐inflammatory dietary patterns rich in fruits, vegetables, whole grains, and healthy fats (Odegaard et al. 2012; Hodge et al. 2018). In our data, frequent TFC aligned with higher DII, indicating elevated preclinical cardiovascular risk; early, scalable strategies that shift both choices and food environments may improve long‐term outcomes.

While health concerns about TF are valid (Saraf et al. 2022; Ntarladima et al. 2022), blanket bans are unlikely to be practical or effective, given enforcement challenges and TF's social and economic roles (Maimaiti et al. 2018; Neckerman 2014). Instead, policy should prioritize targeted, feasible levers that shift choices without eliminating convenience: (i) reformulation targets for sodium, added sugars, and unhealthy fats; (ii) mandatory calorie/sodium labeling on menus and delivery platforms, with prominent warnings for unhealthy items; (iii) choice‐architecture nudges in apps and stores (healthy defaults, prominent placement, search‐ranking that favors healthier options); (iv) procurement standards in schools and public institutions; and (v) calibrated fiscal tools (taxes on unhealthy options paired with subsidies or vouchers for fruits, vegetables, and whole grains). These approaches can reduce CRFs while preserving the accessibility and economic benefits of TFC (Maimaiti et al. 2018; Neckerman 2014; Pacinella et al. 2022; Kamo et al. 2017).

Despite robust associations between TFC, DII, and CRFs, several limitations warrant caution. First, the primary analyses are cross‐sectional, limiting causal inference despite extensive adjustment and leaving room for residual confounding. Second, diet was assessed by a single 24‐h recall; TFC derived from “meals prepared away from home” may misclassify exposure, and DII was computed from a subset of components—measurement error that likely biases estimates toward the null. Third, our survival analyses were secondary and constrained by the 2019 public‐use Linked Mortality Files, a modest number of events, and the absence of adjudicated incident stroke or TOAST subtypes; mortality results should therefore be interpreted conservatively. Fourth, missing data and selection may affect external validity; NHANES is US‐representative, and findings may not generalize internationally. Future work should use repeated dietary measures, adjudicated vascular outcomes, longer follow‐up, and causal designs (prospective cohorts or interventions) to test whether lowering TFC and dietary inflammatory potential improves CRF trajectories and hard outcomes.

## 5 Conclusion

Frequent TFC in US adults was associated with higher energy‐adjusted DII and an adverse cardiometabolic profile (lower HDL; higher triglycerides, fasting glucose, serum insulin, and IR). In survival analyses, higher TFC showed directionally lower survival but no statistically significant associations with all‐cause or heart disease mortality; higher DII was associated with all‐cause mortality and mediated ~1.7% of the TFC–mortality association (not significant). These findings prioritize early, scalable strategies that lower dietary inflammatory potential and curb high‐frequency TFC, especially in younger adults, via education and food‐environment changes. Prospective and interventional studies with repeated diet measures and adjudicated vascular outcomes are needed to test whether reducing TFC and dietary inflammation improves CRF trajectories and hard endpoints.

## Author Contributions

H.W., S.L., and Y.Y. designed research; H.W. and S.L. conducted research; L.X., H.W., and L.M. collected data; M.C. and Z.W. conducted raw data processing; S.L., M.H., and D.Y. analyzed data; T.C.Z. supervised the parameter settings; and H.W. and S.L. wrote the paper. M.Z. and Q.H. had primary responsibility for final content. All authors read and approved the final manuscript.

## Funding

This study was financially supported by Hunan Innovative Province Construction Program (Grant no. XY040018), the Natural Science Foundation of Hunan Province (Grant no. 2019SK2211), Key Research and Development Project of Hunan Province (Grant no. 2020SK2089), and Department of Science and Technology of Hunan Province (Grant no. 2020SK2097). Chen Xiao‐Ping Foundation for the Development of Science and Technology of Hubei Province, 2022 Annual Huaiqi Huang Special Fund for Immunological Disease Research (Grant no. CXPJJH122003‐13).

## Disclosure

The author(s) declare that no Generative AI was used in the creation of this manuscript.

## Ethics Statement

The National Health and Nutrition Examination Survey (NHANES) is an openly accessible dataset in alignment with the endorsement of the National Center for Health Statistics Research Ethics Review Board (NCHS ERB).

## Consent

All of the NHANES participants offered written informed consent.

## Conflicts of Interest

The authors declare no conflicts of interest.

## Supporting information


**Figure S1:** Annual deaths (a) and disability‐adjusted life years (DALYs) (b) from cardiovascular disease (CVD) in the United States, 1990–2021, based on data from the Global Burden of Disease (GBD) database.

**Figure S2:** Age‐Stratified Subgroup Analysis of the Association Between DII and key CRFs, Adjusted for Covariates Including Sex, Race, Educational Attainment, Poverty‐Income Ratio, Weight, Height, Smoking Status, and Physical Activity. DII, Dietary Inflammatory Index; CRFs, Cardiometabolic Risk Factors; FG, fasting glucose; HDL, high‐density lipoprotein; TG, triglycerides; IR, insulin resistance;

**Figure S3:** Sex‐Stratified Subgroup Analysis of the Association Between DII and key CRFs, Adjusted for Covariates Including Age, Race, Educational Attainment, Poverty‐Income Ratio, Weight, Height, Smoking Status, and Physical Activity. DII, Dietary Inflammatory Index; CRFs, Cardiometabolic Risk Factors; FG, fasting glucose; HDL, high‐density lipoprotein; TG, triglycerides; IR, insulin resistance;

**Figure S4:** Age‐Stratified Associations Between TFC, DII, and key CRFs: (a) TFC and DII; (b) TFC and HDL; (c) TFC and TG; (d) TFC and FG; (e) TFC and serum insulin; and (f) TFC and IR; TFC, takeaway food consumption; DII, Dietary Inflammatory Index; CRFs, Cardiometabolic Risk Factors; FG, fasting glucose; HDL, high‐density lipoprotein; TG, triglycerides; IR, insulin resistance;

**Figure S5:** Sex‐stratified associations between TFC and key CRFs: (a) TFC and DII, (b) TFC and HDL, (c) TFC and TG, (d) TFC and FG, (e) TFC and serum insulin, and (f) TFC and IR. TFC, takeaway food consumption; CRFs, Cardiometabolic Risk Factors; DII, Dietary Inflammatory Index; FG, fasting glucose; HDL, high‐density lipoprotein; TG, triglycerides; IR, insulin resistance;

**Figure S6:** Kaplan–Meier survival curves based on TFC quartile groups: (a) All‐cause mortality and (b) Cardiovascular mortality. TFC, Takeaway Food Consumption;

**Figure S7:** Subgroup Analysis of the Associations Between TFC and All‐Cause Mortality by Gender, Race, Education Attainment, Poverty Income Ratio, and Smoking Status. TFC, Takeaway Food Consumption; HR, hazard ratios;

**Figure S8:** Subgroup Analysis of the Associations Between TFC and Cardiovascular Mortality by Gender, Race, Education Attainment, and Smoking Status. TFC, Takeaway Food Consumption; HR, hazard ratios;

**Figure S9:** Associations Between TFC and DII, CMI, and VAI in Subgroup Analyses for All‐Cause and Cardiovascular Mortality. TFC, takeaway food consumption; DII, dietary inflammatory index; CMI, cardiac metabolic index; VAI, visceral adiposity index; HR, hazard ratios;

**Table S1:** Baseline Characteristics of Nutrient Composition of DII Parameters Grouped by TFC Frequency.

**Table S2:** Association of TFC and HDL in 8556 individuals aged 18–80 years: NHANES 2009–2018.

**Table S3:** Association of TFC and TG in 8556 individuals aged 18–80 years: NHANES 2009–2018.

**Table S4:** Association of TFC and FG in 8556 individuals aged 18–80 years: NHANES 2009–2018.

**Table S5:** Association of TFC and serum insulin in 8556 individuals aged 18–80 years: NHANES 2009–2018.

**Table S6:** Association of TFC and IR in 8556 individuals aged 18–80 years: NHANES 2009–2018.

**Table S7:** Association of TFC With All‐Cause and CVD Mortality.

**Table S8:** Associations Between TFC and Pulse, SBP, DBP, TC, LDL, and HbA1c in 8556 Adults Aged 18–80 Years: NHANES 2009–2018.

## Data Availability

The data utilized in this study were sourced from the National Health and Nutrition Examination Survey (NHANES) for the years 2009–2018. Data for the years 2009–2010 were obtained from the NHANES website (https://wwwn.cdc.gov/nchs/nhanes/continuousnhanes/default.aspx?BeginYear=2009), while data for the years 2011–2012 were acquired from the NHANES website (https://wwwn.cdc.gov/nchs/nhanes/continuousnhanes/default.aspx?BeginYear=2011), while data for the years 2013–2014 were acquired from the NHANES website (https://wwwn.cdc.gov/nchs/nhanes/continuousnhanes/default.aspx?BeginYear=2013), while data for the years 2015–2016 were acquired from the NHANES website (https://wwwn.cdc.gov/nchs/nhanes/continuousnhanes/default.aspx?BeginYear=2015), while data for the years 2017–2018 were acquired from the NHANES website (https://wwwn.cdc.gov/nchs/nhanes/continuousnhanes/default.aspx?BeginYear=2017). The availability and accessibility of these datasets were ensured through the NHANES data repository.
