# Supplementary figures and images for: Takeaway Food Consumption, Dietary Inflammatory Index, and Cardiometabolic Risk Factors in US Adults: Findings From NHANES (2009–2018)
Source: Food Sci Nutr. 2025 Dec 9;13(12):e71316. doi: 10.1002/fsn3.71316 (PMC12689940; doi:10.1002/fsn3.71316)

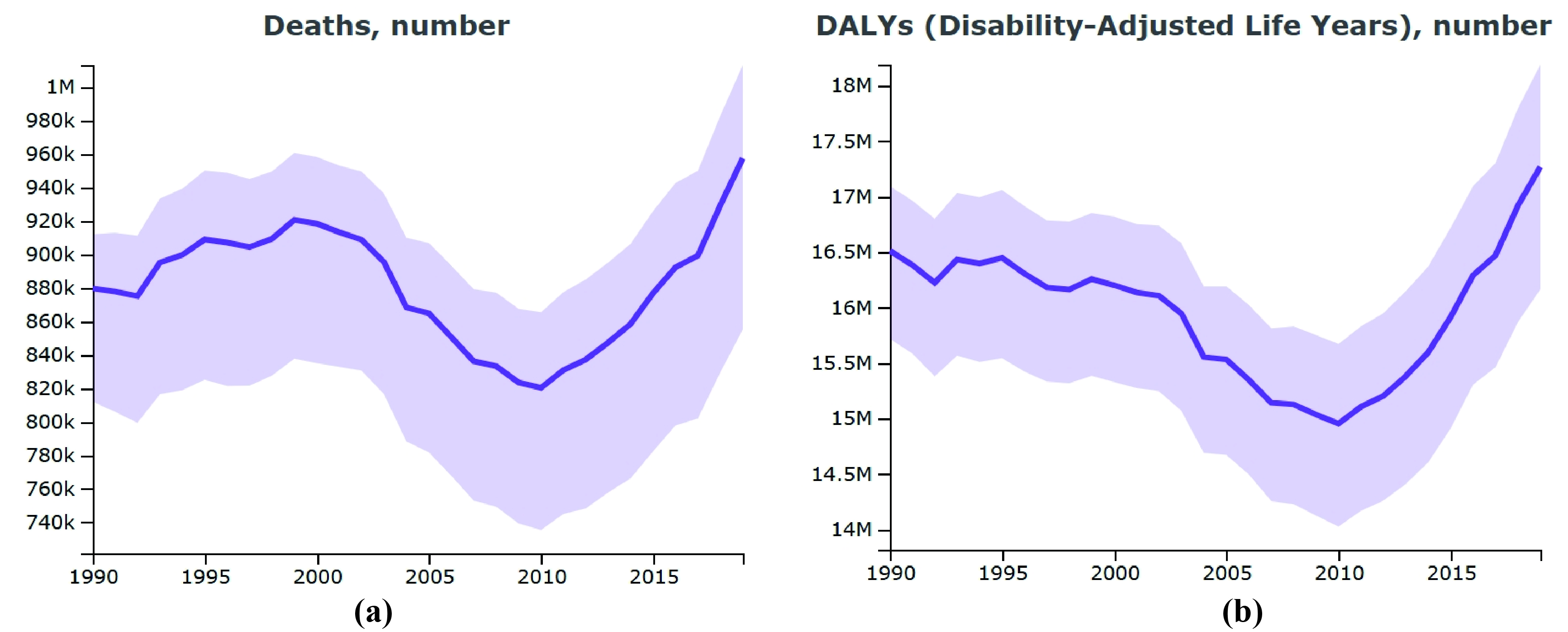

Supplement: Supplementary file 1 — Figure S1: Annual deaths (a) and disability‐adjusted life years (DALYs) (b) from cardiovascular disease (CVD) in the United States, 1990–2021, based on data from the Global Burden of Disease (GBD) database. [file FSN3-13-e71316-s006.jpg]

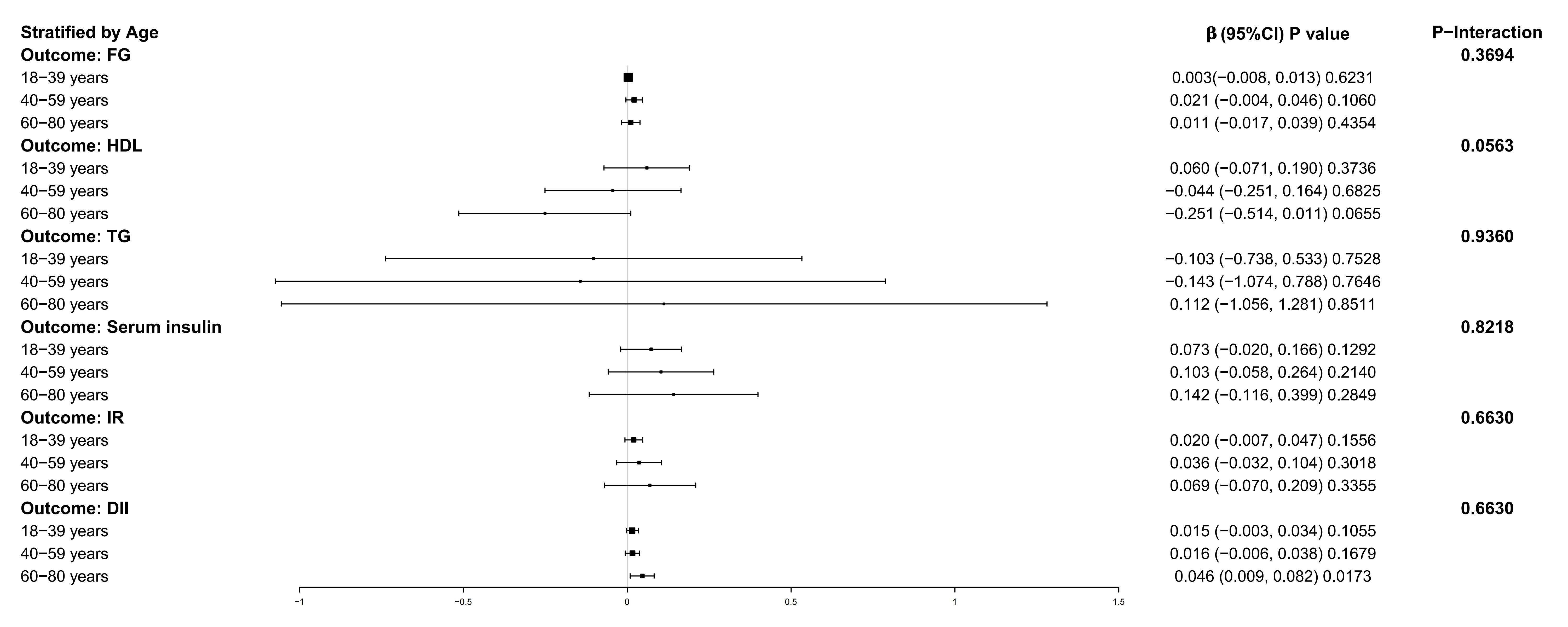

Supplement: Supplementary file 2 — Figure S2: Age‐Stratified Subgroup Analysis of the Association Between DII and key CRFs, Adjusted for Covariates Including Sex, Race, Educational Attainment, Poverty‐Income Ratio, Weight, Height, Smoking Status, and Physical Activity. DII, Dietary Inflammatory Index; CRFs, Cardiometabolic Risk Factors; FG, fasting glucose; HDL, high‐density lipoprotein; TG, triglycerides; IR, insulin resistance; [file FSN3-13-e71316-s003.jpg]

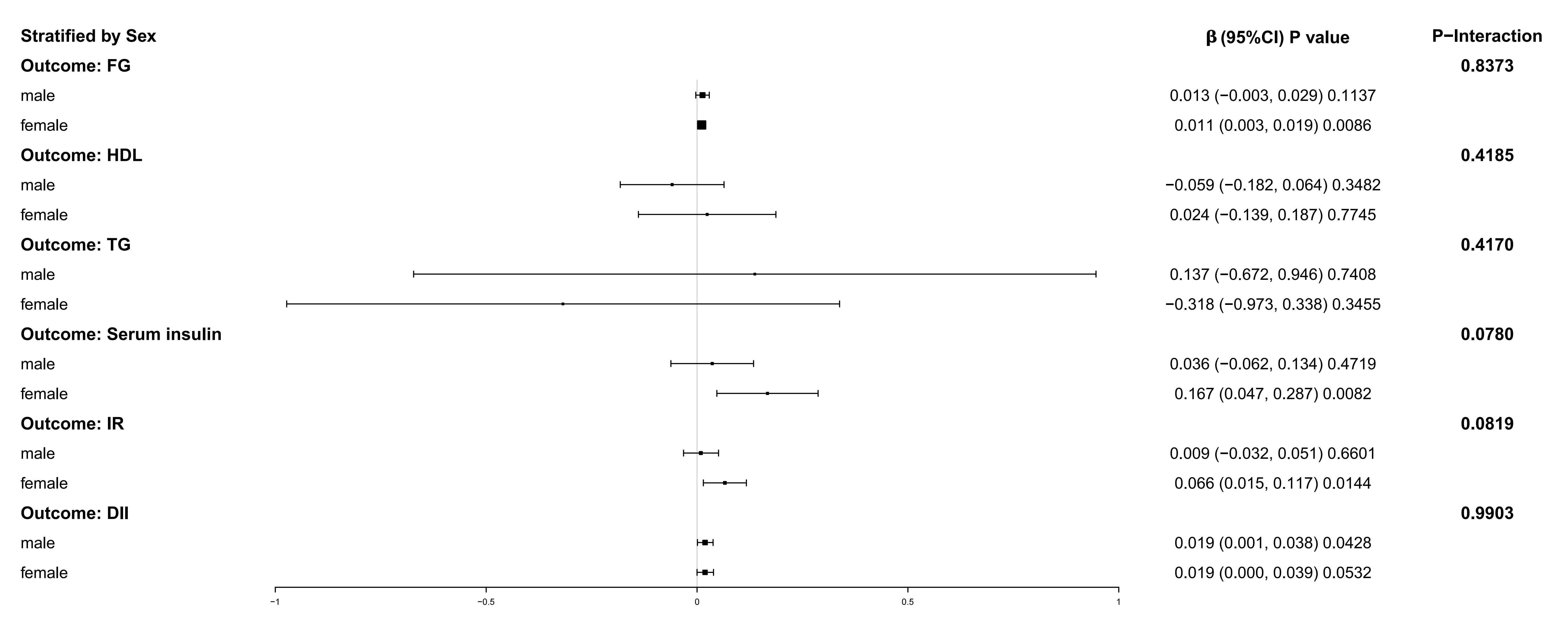

Supplement: Supplementary file 3 — Figure S3: Sex‐Stratified Subgroup Analysis of the Association Between DII and key CRFs, Adjusted for Covariates Including Age, Race, Educational Attainment, Poverty‐Income Ratio, Weight, Height, Smoking Status, and Physical Activity. DII, Dietary Inflammatory Index; CRFs, Cardiometabolic Risk Factors; FG, fasting glucose; HDL, high‐density lipoprotein; TG, triglycerides; IR, insulin resistance; [file FSN3-13-e71316-s014.jpg]

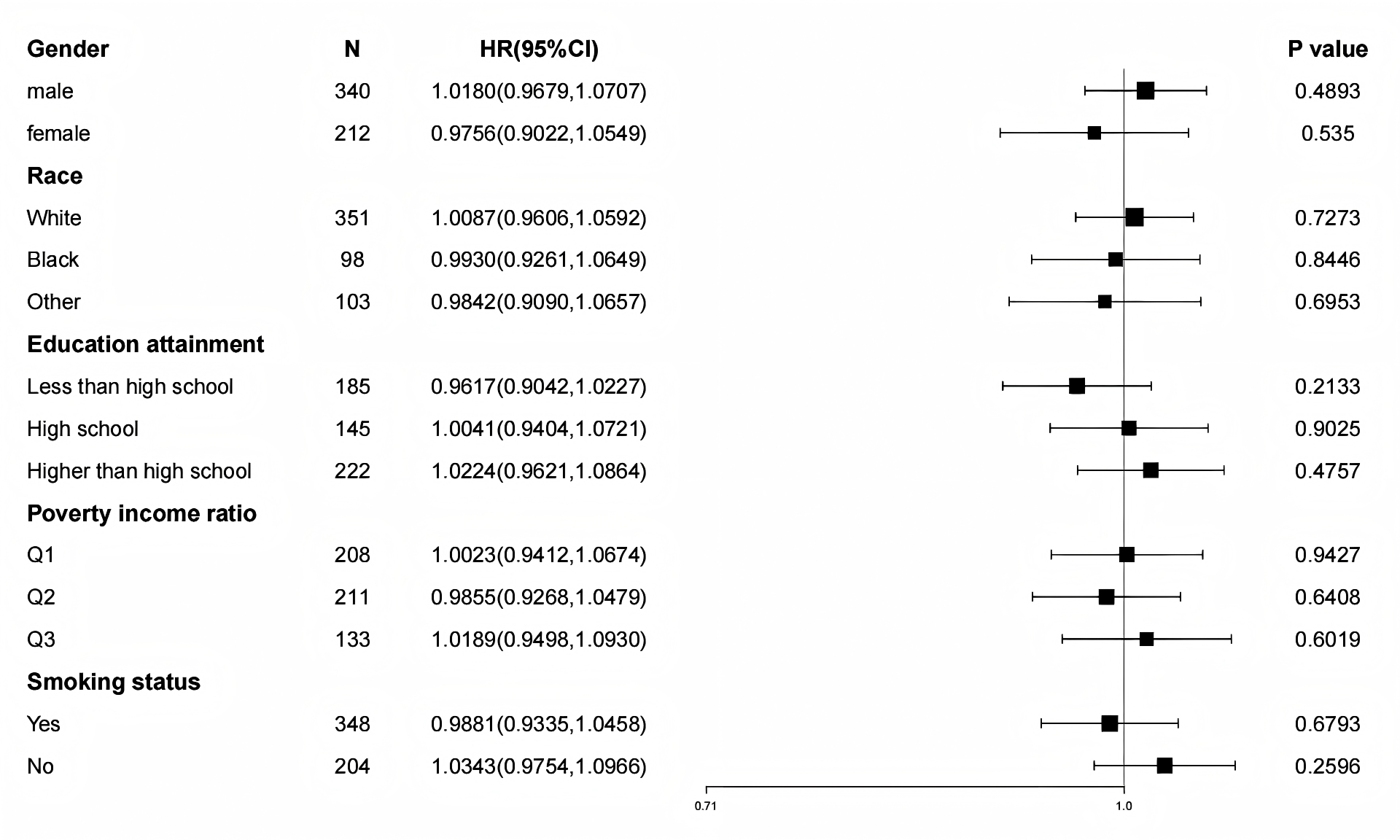

Supplement: Supplementary file 7 — Figure S7: Subgroup Analysis of the Associations Between TFC and All‐Cause Mortality by Gender, Race, Education Attainment, Poverty Income Ratio, and Smoking Status. TFC, Takeaway Food Consumption; HR, hazard ratios; [file FSN3-13-e71316-s013.png]

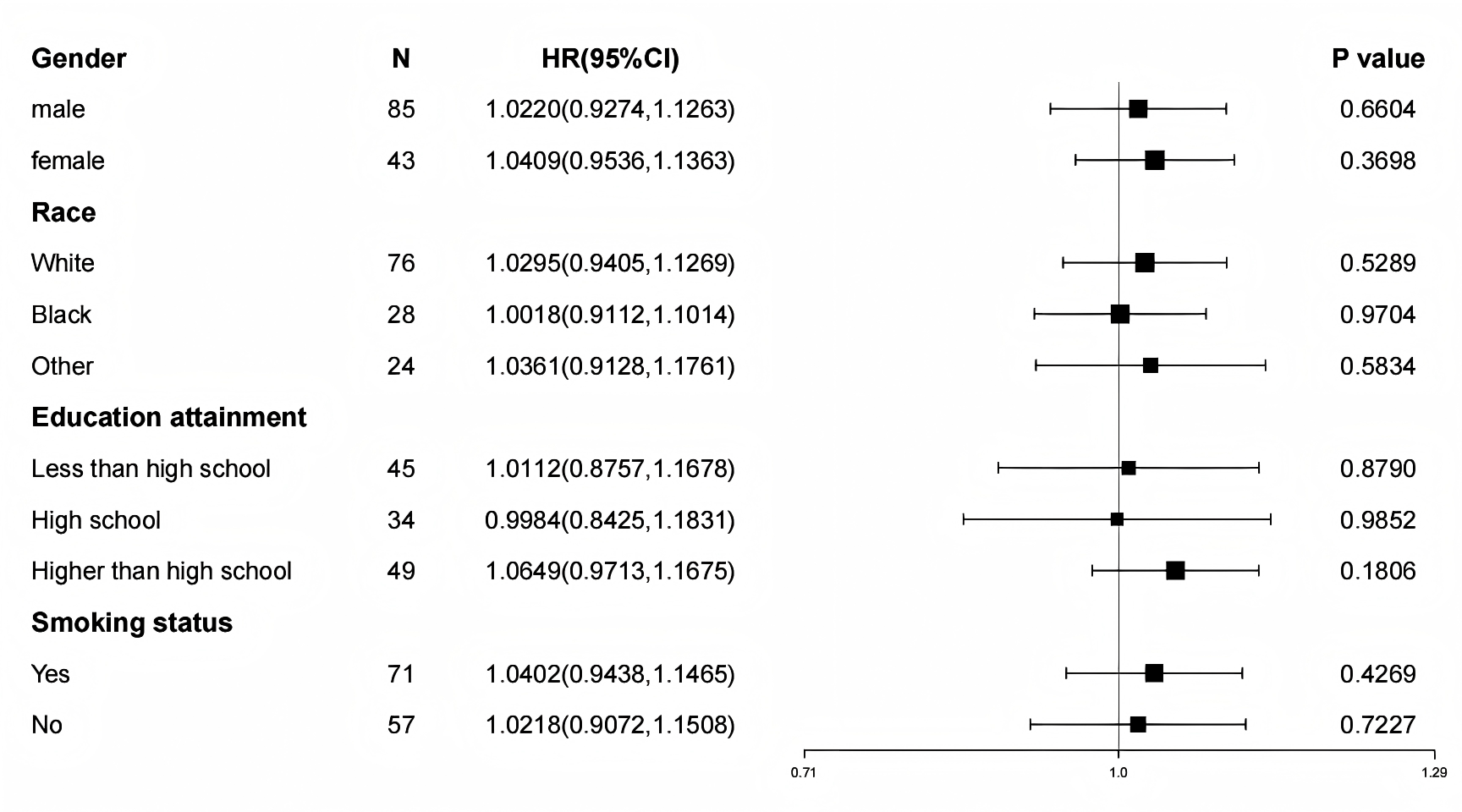

Supplement: Supplementary file 8 — Figure S8: Subgroup Analysis of the Associations Between TFC and Cardiovascular Mortality by Gender, Race, Education Attainment, and Smoking Status. TFC, Takeaway Food Consumption; HR, hazard ratios; [file FSN3-13-e71316-s001.png]

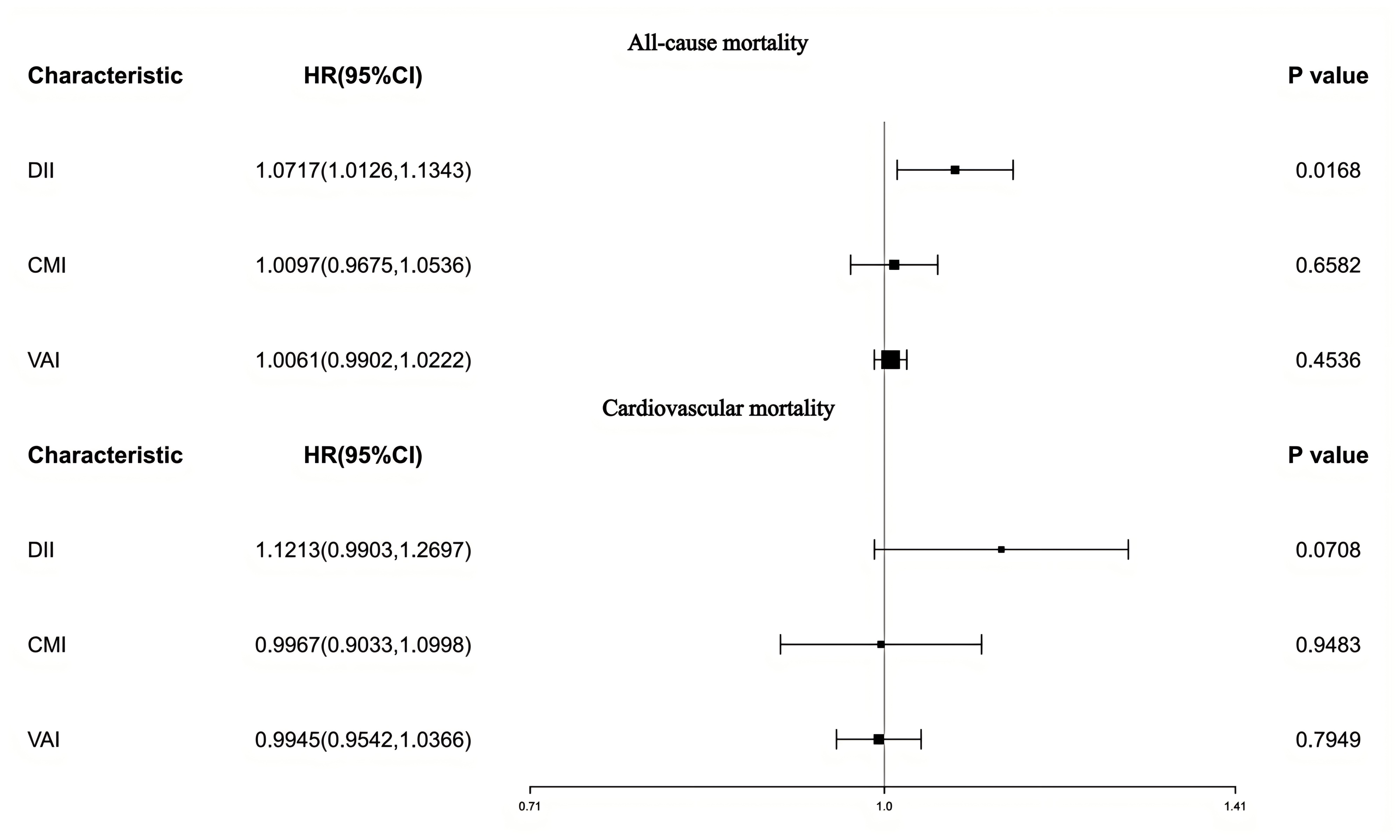

Supplement: Supplementary file 9 — Figure S9: Associations Between TFC and DII, CMI, and VAI in Subgroup Analyses for All‐Cause and Cardiovascular Mortality. TFC, takeaway food consumption; DII, dietary inflammatory index; CMI, cardiac metabolic index; VAI, visceral adiposity index; HR, hazard ratios; [file FSN3-13-e71316-s012.png]
